# Rapid and Sensitive Liquid Chromatographic Method for Determination of Paclitaxel from Parenteral Formulation and Nanoparticles

**DOI:** 10.4103/0250-474X.73914

**Published:** 2010

**Authors:** S. Kollipara, G. Bende, R. N. Saha

**Affiliations:** Formulation Development and Pharmacokinetics Laboratory, Pharmacy Group, Birla Institute of Technology and Science, Pilani-333 031, India

**Keywords:** nanoparticles, paclitaxel, parenteral formulation, RP-HPLC

## Abstract

A simple and fast reversed phase liquid chromatographic method was developed for estimation of paclitaxel in commercially available parenteral formulation and nanoparticles. Separations were carried out using mobile phase consisting of acetonitrile and 20 mM potassium dihydrogen phosphate (45:55, v/v) on Lichrocart^®^ C_18_ analytical column at a flow rate of 1 ml/min and detection wavelength of 230 nm. The developed method exhibited linearity over an analytical range of 50-2000 ng/ml with regression equation, mean peak area= 137.58 concentration (ng/ml)+1765.94, (R_2_ =0.9999). The method demonstrated selectivity with no interfering peaks eluting near the vicinity of drug peak. The method was found to be sensitive with detection and quantification limits of 7.57 ng/ml and 22.94 ng/ml. The method has shown consistent and good recoveries from parenteral formulation (100.06±0.86%) and nanoparticles (100.43±0.91%). The method was successfully employed for the analysis of *in vitro* release study samples of nanoparticle formulation. The method was also applied for determination of paclitaxel content in various pharmaceutical formulations.

Paclitaxel (PAC) is a potent chemotherapeutic agent ((1S,2S,3R,4S,7R,9S,10S,12R,15S)-4,12-diacetoxy-15-{[(2R,3S)-3-(benzoylamino)-2-hydroxy-3-phenylpropanoyl] oxy}-1,9-dihydroxy-10,14,17,17-tetramethyl-11-oxo-6-oxatetracyclo[11.3.1.0~3,10~.0~4,7~] heptadec-13-en-2-yl rel-benzoate) indicated as first-line and subsequent therapy for treatment of lung, ovarian, breast, neck cancers and solid tumors[1]. PAC binds to β-subunit of tubulin and subsequently hyper-stabilizes its structure. This leads to disruption of normal microtubule dynamics and as a consequence causes cell death. Other mechanism of action has also been proposed, in which PAC induces apoptosis in tumor cells by binding to an apoptosis stopping protein called Bcl-2 (B-cell leukemia 2). PAC is a high molecular weight drug (M_w_ 845 Da) with very limited aqueous solubility (<30 µg/l) and high hydrophobicity (log P 3.96)[2]. *In vitro* studies in human liver microsomes and tissue slices showed majority of administered PAC dose was metabolized primarily by CYP2C8 and CYP3A4. Due to its poor physicochemical properties, affinity for metabolizing enzymes (CYP2C8, CYP3A4) and efflux transporters (P-glycoprotein), the oral bioavailability of PAC is found to be less than 10%[3]. These limitations led to the development of non-aqueous intravenous injection (Taxol^®^, containing 6 mg/ml of PAC), in which PAC is dissolved in a media containing (1:1, *v/v*) mixture of polyoxyethylated castor oil (Cremophor EL) and dehydrated alcohol. This solution is to be diluted with a suitable parenteral fluid before intravenous infusion. However there have been serious complications associated with the use of Taxol^®^ like development of hypersensitivity reactions, lethargy, hypotension, neurotoxicity, nephrotoxicity, vasodilation, attributed to the presence of Cremophor EL[4]. In order to develop a safe and effective formulation for controlled delivery of PAC, nanoparticles have been prepared in our laboratory.

Literature survey revealed many liquid chromatography based methods for estimation of PAC in biological fluids[5–8]. Very few methods were reported for estimation of PAC from pharmaceutical dosage forms[9,10]. However, the developed methods used complex gradient systems and longer analysis time making them unsuitable for routine analysis. In the present work, a fast, sensitive and economic reversed phase liquid chromatographic method was developed for estimation of PAC in bulk, nanoparticles, parenteral formulations, and for *in vitro* release study of nanoparticle formulations. The developed method was validated as per ICH guidelines and applied successfully for determination of PAC content from commercially available parenteral formulation and in-house developed nanoparticles.

## MATERIALS AND METHODS

Paclitaxel (Assay 99.95%) obtained as a gift sample from Dr. Reddy’s Laboratories, Hyderabad, India. HPLC grade acetonitrile, dichloromethane were procured from Spectrochem, India. Analytical grade potassium dihydrogen phosphate was purchased from S. D. Fine Chemicals Ltd., Mumbai, India. Poly(lactic-co-glycolic acid) copolymer (PLGA) was generously gifted as a test sample by Purac, USA. Poly vinyl alcohol was procured from Sigma-Aldrich Chemicals Ltd., St. Louis MO, USA. Miglyol 810 was purchased from Sasol Chemicals, Germany. Taxol^®^ (5 ml vial, Bristol-Myer’s Sqibb) was obtained from the local pharmacy. Ultra pure water was prepared using a Milli-Q^®^ water purification system (Millipore Co., USA) and filtered (0.22 µm) before use. All other chemicals used in the study were of analytical grade.

### Chromatographic system and conditions:

The HPLC system (Jasco, Japan) used in present study consisted of PU-1580 pump, AS-1559 auto injector and UV-1575 UV/Vis detector. Separations were carried out on Lichrocart^®^ RP_18_ reversed phase column (Merck^®^, 120×4.6 mm; 5 µm). Chromatographic peaks were integrated using Borwin^®^ work station (Jasco, Japan) loaded on a computer system (IBM, USA). Optimized mobile phase was prepared by mixing acetonitrile and 20 mM potassium dihydrogen phosphate buffer in 45:55 (%, *v/v*) ratio and degassed in an ultrasonic bath for 15 min just before chromatography. The flow rate was set to 1 ml/min. Injection volume of 50 µl was made and the column eluents were monitored at 230 nm over a run time of 10 min. All the separations were carried out at ambient conditions (25°) after baseline stabilization for at least 30 min.

### Preparation of nanoparticles:

Nanoparticles of PAC were prepared by emulsion solvent evaporation technique[11]. Briefly, 10 mg of PLGA, 35 µl of miglyol, 5 mg of PAC were dissolved in 1 ml of methylene chloride. This organic phase was dispersed in 10 ml aqueous phase containing poly vinyl alcohol (2%, *w/v*) as stabilizer. The resulting emulsion was stirred on magnetic stirrer over night for complete evaporation of organic phase. The preparation was centrifuged at 14 000 rpm at 25° for 30 min (Remi Compufuge, India). The nanoparticle containing fraction was washed twice with phosphate buffered saline and freeze dried (Maxi Dry Lyo, Heto, Germany) over a period of 10 h. Similarly placebo nanoparticles were also prepared without adding PAC.

### Preparation of stocks and standards:

Primary stock solution of 1 mg/ml of PAC was prepared in acetonitrile. Secondary stock solution of 10 µg/ml was prepared by diluting 1 ml of primary stock to 100 ml using solvent media consisting of acetonitrile: MilliQ^®^ water (50:50, *v/v*). Three separate series of six calibration standards 50, 100, 250, 1000 and 2000 ng/ml were prepared by serial dilution in mobile phase. Sample standards of PAC were prepared by adding known amount of drug in blank nanoparticles and placebo mixture of parenteral formulation at five levels: 25, 50, 100, 150, 200% of the labeled claim. Similarly, placebo standards were also prepared without adding PAC. The sample standards and placebo standards were processed independently using their respective sample preparation method as described below.

### Sample preparation:

For parenteral formulation, 100 µl of placebo/sample standard or test sample was added to 10 ml volumetric flask and the volume was made up with acetonitrile. The sample was vortex mixed and 0.83 ml of sample was added to 100 ml volumetric flask and diluted to volume with mobile phase.

For nanoparticles, a quantity of formulation (placebo/sample standard or test sample) equivalent to 2 mg of PAC was weighed and transferred to a 10 ml volumetric flask. The nanoparticles were digested with 2 ml acetonitrile by ultra-sonication (10 min, 25°). The volume was made with acetonitrile and the samples were centrifuged at 10 000 rpm for 15 min. Finally, 0.25 ml of supernatant was transferred to 100 ml volumetric flask and the volume was made with mobile phase.

### Analytical method validation:

The developed reversed phase chromatographic method was validated for selectivity, linearity, range, precision, accuracy, sensitivity and system suitability as per ICH guidelines[12]. The proposed method was also applied for drug content analysis from parenteral formulation and in-house prepared nanoparticles.

### Selectivity:

The selectivity of the method in presence of formulation excipients was assessed by injecting the processed placebo and sample standards in three triplicates on three different days. The obtained chromatograms were compared with freshly prepared calibration standards.

### Linearity and range:

The linearity of the method was assessed by analyzing the calibration standards in three replicates on three different days. Average peak area was plotted against respective concentration level and subjected to least square linear regression. Calibration curve obtained from regression analysis was used to calculate the corresponding predicted concentrations. The analytical range of proposed method was obtained by analyzing residuals. One-way analysis of variance (ANOVA) was performed on each replicate obtained at six concentration levels[13].

### Accuracy and precision:

Placebo spiking technique was employed for establishing accuracy of the method. Sample standards containing 25, 50, 100, 150, 200% of the labeled claim of parenteral formulation and nanoparticles were processed in five replicates and injected on three different days. The results were expressed as mean absolute recovery, % bias and coefficient of variation (%CV).

Precision of the method was expressed as repeatability (intra-batch) and intermediate precision (inter-batch). For repeatability, five series of quality control (QC) samples prepared at lower (LQC, 50 ng/ml), middle (MQC, 500 ng/ml) and higher (2000 ng/ml) were freshly prepared and analyzed. For intermediate precision, five series of quality control samples were prepared and analyzed on three different days. The results of precision were expressed as %CV.

### Sensitivity:

The sensitivity of proposed method was determined using standard deviation of intercept (σ) and slope (s) of calibration equation. Limit of detection (LOD) and limit of quantification (LOQ) were calculated using 3.3 σ/s and 10 σ/s respectively.

### System suitability and sample solution stability:

System precision was carried out by analyzing six replicates of calibration standards. Various chromatographic parameters like capacity factor (k), tailing factor (T_f_) and number of theoretical plates (N) were recorded. Further, the stability of PAC in mobile phase was determined by injecting calibration standard 1000 ng/ml at 0, 6, 12, 24, 36, and 48 h in five replicates.

### Formulation analysis:

As an application, the proposed method was used for determination of drug content from marketed formulation Taxol^®^ and in-house prepared nanoparticles. For Taxol^®^, 100 µl of sample was taken and processed as in sample preparation section. For nanoparticles, amount equivalent to 2 mg of PAC was accurately weighed and processed as described in sample preparation section. Finally, 50 µl of resulting solution was injected in triplicates and analyzed.

### *In vitro* release study of nanoparticles:

The *in vitro* release study of nanoparticles was carried out in triplicates using dialysis bag diffusion method using phosphate buffer saline (pH 7.4) with Tween 80 (2%, *w/v*) as dissolution media[14]. Briefly, 5 mg of freeze dried nanoparticles were dispersed in 2 ml of MilliQ^®^ water and placed in a dialysis membrane bag (Molecular weight cutoff 12 KDa) and sealed. Release studies were carried out using modified USP Type II apparatus (Electrolab, India) with 50 ml of dissolution media, set at 50 rpm, 37±2°. Samples of 2 ml were withdrawn at specified time points over a period of 48 h and same amount of blank dissolution media was added. The obtained samples were centrifuged and analyzed by proposed HPLC method. The obtained *in vitro* release data was fitted into various mathematical models like zero order, first order, Higuchi model and reciprocal powered time (RPT) model[15].

## RESULTS AND DISCUSSION

The aim of the present study was to develop a simple, accurate and precise reversed phase HPLC method for quantification of PAC in bulk and in pharmaceutical formulations. Based on the UV-profile of PAC, the wavelength was optimized at 230 nm for better sensitivity and selectivity from formulation excipients. Initial optimization of mobile phase was carried out to select a suitable organic modifier and buffering agent. Initial trials with acetonitrile and MilliQ water (50:50, *v/v*) resulted in poor peak parameters with inadequate retention (Rt ≈ 3.5 min). Trials with acetonitrile and 20 mM potassium dihydrogen phosphate buffer (50:50, *v/v*) showed better peak parameters with improved retention (Rt ≈ 4.7 min). Based on above results, the final mobile phase was set to acetonitrile and 20 mM potassium dihydrogen phosphate buffer (45:55, *v/v*). This combination of mobile phase showed excellent chromatographic peak parameters with good retention (Rt 7.7±0.2 min).

**Fig. 1:**
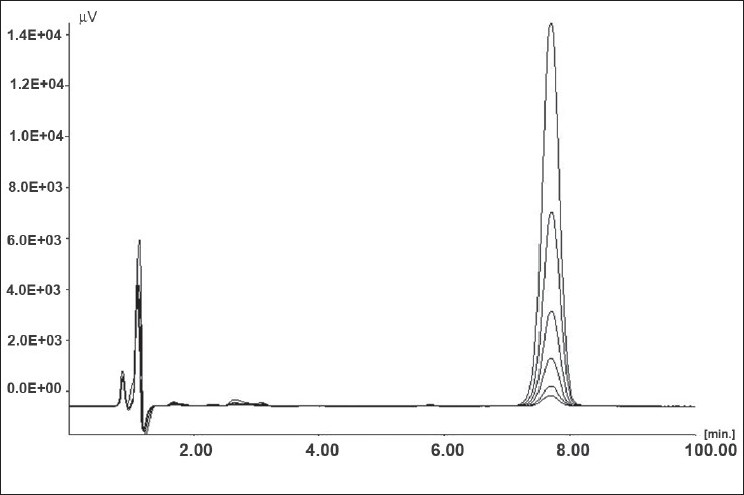
Overlay of calibration standards (50-2000 ng/ml).

**Fig. 2:**
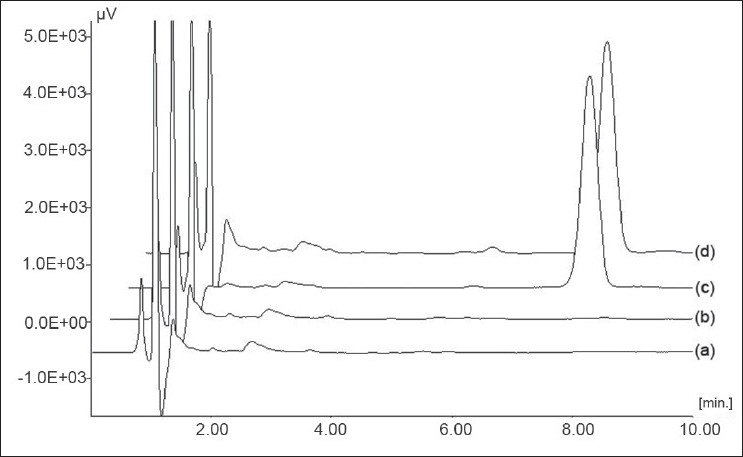
Chromatograms of standards (a) placebo standard for parenteral formulation, (b) placebo standard for nanoparticles, (c) sample standard (100%) for parenteral formulation, (d) sample standard (100%) for nanoparticles

Placebo standards showed no interference in the vicinity of PAC peak, when compared with freshly prepared calibration standards indicating the selectivity of developed method for PAC in presence of formulation excipients. Overlay of calibration standards is shown in fig. 1 and overlay of placebo standards and sample standards (100%) is shown in fig. 2.

Calibration data of PAC is shown in Table 1. The calibration curve obtained by least square analysis showed linear relationship with regression coefficient (R^2^) of 0.9999. The best fit equation obtained was mean peak area= 137.58×concentration (ng/ml) + 1765.94. At all concentration levels, the standard deviation was low and %CV did not exceed 2%. The predicted concentrations were in close agreement with the theoretical concentrations. The linearity range was found to be 50-2000 ng/ml. Analysis of residuals showed that the residuals were normally distributed with uniform variance across studied concentration levels, indicating homoscedastic nature of the data. The standard error of slope and intercept were found to be 0.150 and 140.883, respectively. The obtained slope and intercept values were well within the 95% confidence intervals (confidence interval for slope: 137.16-137.99, confidence interval for intercept: 1374.78-2157.09). The goodness of fit of linear regression equation was supported by low standard error or estimate (203.95, with respect to mean peak area and 1.48 with respect to concentration). The one-way ANOVA performed on peak area at each concentration level indicated that calculated F-value (0.22×10^-3^) was less than the critical F-value (2.152) at 5% significance level.

**TABLE 1:** CALIBRATION DATA FOR PACLITAXEL

Concentration (ng/ml)	Average peak area a (±SD)	%CV	Predicted concentration (ng/ml)
50	8498.00 ± 62.11	0.73	48.93
100	15456.67 ± 190.50	1.23	99.51
250	36462.67 ± 282.83	0.78	252.20
500	70308.89 ± 518.60	0.74	498.22
1000	139592.56 ± 1515.72	1.09	1001.82
2000	276822.67 ± 2618.24	0.95	1999.31

a each value represents the average of nine independent determinations

The developed method showed high and consistent absolute recoveries at all studied levels for both parenteral formulation and nanoparticles. The recovery data from parenteral formulation and nanoparticles is shown in Table 2. For parenteral formulation, the mean absolute recovery ranged from 99.61 to 100.60% and for nanoparticles, it ranged from 100.23 to 100.75%. At all studied concentration levels, the standard deviation was low (< 1.40) representing the accuracy of the proposed method. Additionally, the obtained recoveries were found to be normally distributed with low and uniform %CV at all concentration levels. Moreover, the %bias values were low (< 1) at all studied levels indicating that there was no significant interference of formulation excipients. Hence, the recovery study demonstrated the suitability of proposed method for determination of PAC from parenteral formulation and nanoparticles.

**TABLE 2:** RECOVERY STUDY BY PLACEBO SPIKING TECHNIQUE

Product	Amount of drug added (% of label claim)^a^	Mean absolute recovery (%)	%CV	% Bias
Parenteral Formulation	25	100.27±0.67	0.66	0.27
	50	100.60±1.31	1.30	0.60
	100	99.85±0.50	0.50	0.15
	150	99.61±0.82	0.82	0.39
	200	99.96±0.57	0.57	0.04
	Overall recovery = 100.06 ± 0.86			
Nanoparticles	25	100.54±0.71	0.71	0.71
	50	100.41±1.15	1.15	1.15
	100	100.23±1.01	1.01	1.01
	150	100.24±0.82	0.82	0.82
	200	100.75±0.91	0.91	0.91
	Overall recovery = 100.43±0.91			

The results for precision study are shown in Table 3. In repeatability, the %CV ranged from 0.16 to 2.06. At all QC levels, the variation was insignificant indicating the repeatability of the method. Similarly, low inter-batch %CV (< 1.83) was observed for intermediate precision. The %CV values were very well within the acceptable range indicating the repeatability and intermediate precision of the developed method.

**TABLE 3:** RESULTS FOR REPEATABILITY AND INTERMEDIATE PRECISION

QC Level	Repeatability (intra-batch)						Intermediate precision	
	Batch-I		Batch-II		Batch-III		(inter-batch)	
	Mean a	%CV	Mean a	%CV	Mean a	%CV	Mean	%CV
LQC	49.91	1.82	50.27	1.87	50.38	2.06	50.19	1.83
MQC	501.62	0.97	499.28	1.04	499.90	1.13	500.27	0.99
HQC	2022.48	0.23	2014.32	0.27	2011.28	0.16	2016.03	0.32

a each value represents average of five independent determinations

The LOD and LOQ of the method were found to be 7.57 and 22.94, ng/ml respectively. The method has demonstrated high value of slope with minimal standard error. Insignificant change in chromatographic peak properties (retention time and peak area) were observed upon re-injection at quantification limit. Hence the method was found to be highly sensitive for determination of PAC.

The method has shown excellent chromatographic peak parameters such as capacity factor (k ≥ 3.25), number of theoretical plates (N≥3700) and tailing factor (T_f_ ≈1.065). The obtained peak parameters were well within the acceptable limits indicating the suitability of the method for PAC determination. Low variability in peak area and retention time were observed upon re-injection indicating that the developed method was specific, precise and stable for estimation of PAC. In addition, the drug peak exhibited no response and chromatographic change for 48 h when compared against freshly prepared standards. The results demonstrated the stability of drug in mobile phase over a period of 48 h with variation less than 0.80%.

The developed method was used to estimate the PAC content from marketed formulation Taxol^®^ and in-house prepared nanoparticles. The mean recoveries for each formulation were in good agreement with the labeled claim indicating the accuracy of method for determination of PAC. The mean absolute recovery for Taxol^®^ and nanoparticle formulation were found to be 100.79 and 99.52% respectively. The %CV was found to be 0.81 and 1.85% for Taxol^®^ and nanoparticles, respectively. Thus, the proposed method was found to be suitable for determination of PAC from both the formulations.

**Fig. 3:**
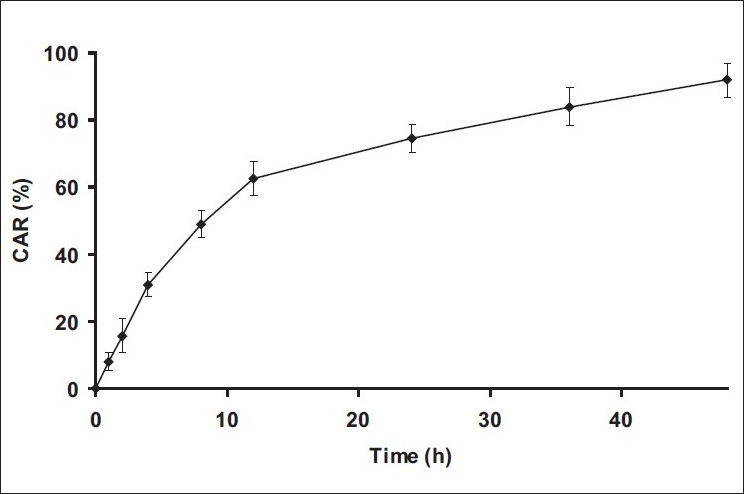
*In vitro* release study of nanoparticles CAR is cumulative amount released, mean±SD, n=3

The developed method was applied successfully to determine amount PAC from *in vitro* release study. The *in vitro* release profile of PAC from nanoparticles is shown in fig. 3. The release of PAC from nanoparticles showed initial burst till 12 h followed by continuous and slow release till 48 h. The release profile was also evaluated by fitting into different mathematical models. The release profile was described better by RPT model (R^2^ =0.9913) compared to zero order (R^2^ =0.8228), first order (R^2^ =0.9790) and Higuchi model (R^2^ =0.9597). The time for 50% dissolution (t_50%_) was found to be 8.02 h.

In summary, the proposed method was found to be simple, sensitive, accurate, and precise for estimation of PAC from parenteral formulation and nanoparticles. The analysis of parenteral and in-house prepared nanoparticle formulations showed good agreement with labeled claims indicating insignificant interference of formulation excipients in the estimation. The method was also successfully applied for determination of amount of PAC release from nanoparticles. Thus, the method can be used for routine analysis of PAC from bulk and pharmaceutical formulations.
